# Regulation of Sixth Seminal Root Formation by Jasmonate in *Triticum aestivum* L.

**DOI:** 10.3390/plants10020219

**Published:** 2021-01-23

**Authors:** Alexey Pigolev, Dmitry Miroshnichenko, Sergey Dolgov, Tatyana Savchenko

**Affiliations:** 1 Institute of Basic Biological Problems, Pushchino Scientific Center for Biological Research, Russian Academy of Sciences, 142290 Pushchino, Russia; alexey-pigolev@rambler.ru; 2 Branch of Shemyakin and Ovchinnikov Institute of Bioorganic Chemistry, Russian Academy of Sciences, 142290 Pushchino, Russia; miroshnichenko@bibch.ru (D.M.); dolgov@bibch.ru (S.D.)

**Keywords:** wheat, *Triticum aestivum*, seminal roots, root initiation, jasmonates, exogenous application

## Abstract

A well-developed root system is an important characteristic of crop plants, which largely determines their productivity, especially under conditions of water and nutrients deficiency. Being Poaceous, wheat has more than one seminal root. The number of grown seminal roots varies in different wheat accessions and is regulated by environmental factors. Currently, the molecular mechanisms determining the number of germinated seminal roots remain poorly understood. The analysis of the root system development in germinating seeds of genetically modified hexaploid wheat plants with altered activity of jasmonate biosynthesis pathway and seeds exogenously treated with methyl jasmonate revealed the role of jasmonates in the regulation of sixth seminal root development. This regulatory effect strongly depends on the jasmonate concentration and the duration of the exposure to this hormone. The maximum stimulatory effect of exogenously applied methyl jasmonate on the formation of the sixth seminal root was achieved at 200 μM concentration after 48 h of treatment. Further increase in concentration and exposure time does not increase the stimulating effect. While 95% of non-transgenic plants under non-stress conditions possess five or fewer seminal roots, the number of plants with developed sixth seminal root reaches up to 100% when selected transgenic lines are treated with methyl jasmonate.

## 1. Introduction

The root system of mature wheat plants is represented by embryonic and postembryonic roots. Embryonic (or seminal) roots are formed during embryogenesis, emerge at the very early stages of plant development, and initially grow using nutrients from endosperm. The average number of roots in 7-day-old wheat plants can vary from 2.5 to 6.45 [1]. The primordium for five seminal roots can be easily distinguished in embryos, while the small size and position of the sixth primordium make it very difficult to distinguish, or this primordium may even not be formed due to the earlier cessation of embryo development [2,3,4,5]. To the best of our knowledge, the possible primordium for the sixth seminal root has been demonstrated only in one study [3]. Not all primordia develop into roots, and the number of developed seminal roots depends on the environmental conditions [6,7,8,9]. Wild wheat species usually develop only three seminal roots, even though the primordia for five seminal roots can be clearly observed in the embryo [2]. The other two primordia may not grow at all or grow only after an episode of drought, in case of damage or death of the first three seminal roots. The sixth seminal root rarely emerges under normal growth conditions, but grow in 55% of the seedlings during recovery from dehydration stress [2,10]. Domesticated wheat species, including the most cultivated durum wheat (*Triticum durum*) and bread wheat (*T. aestivum*), often grow five roots at the very early stages of seedling development, although in general this parameter is quite variable, particularly in hexaploid accessions. *Triticum durum* cultivars more tend to have six roots [2,11]. It was suggested that the number of seminal roots negatively correlates with the primary seminal root length over the course of cultivars evolution [12].

**Figure 1:**
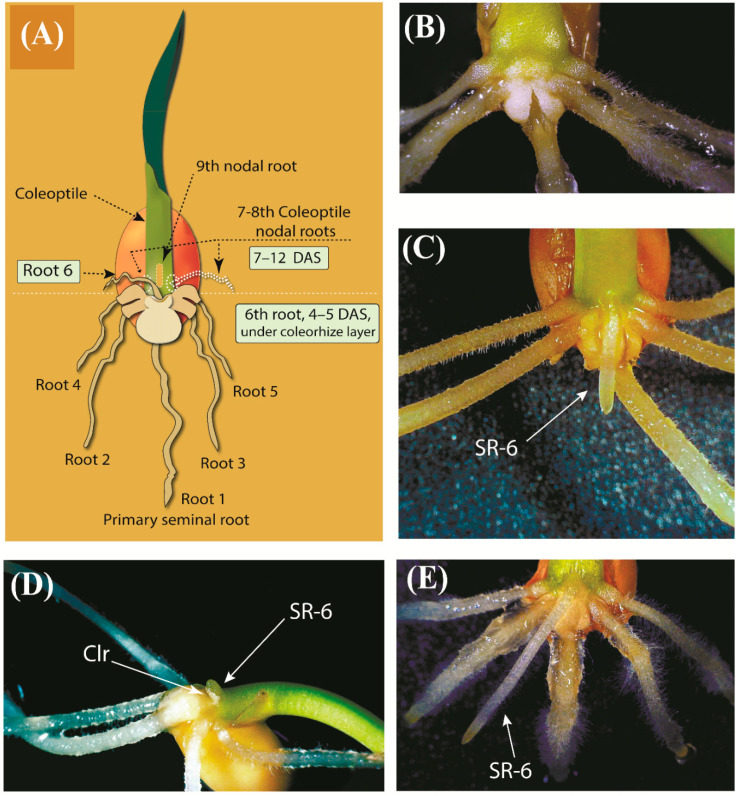
General characteristic features of the sixth seminal root (SR-6). (**A**) A schematic representation of a young wheat seedling, illustrating the position of six seminal roots and three coleoptile nodal roots (the latter are shown with dotted lines); (**B**–**E**) images of germinating ‘Saratovskaya-60’ (Sar-60) seeds: seedling at tenth day after sowing (DAS) with five developed seminal roots without SR-6 (**B**); seedling at sixth DAS with SR-6 (**C**); the beginning of the SR-6 development accompanied by a characteristic displacement of coleorhiza tissue (**D**); (**E**) a plant treated with 500 μM methyl jasmonate (MeJA) with six seminal roots abundantly covered with root hairs, wherein SR-6 length is similar to that of other seminal roots.

The primary seminal root emerges through the coleorhiza within one or two days after imbibition, thereby initiating the formation of the root system, then the first pair of seminal roots appears within 1–4 days (Figure 1). One to four days later, another pair of seminal roots appears above the first pair. The 6th seminal root may appear in 5 to 10 days after imbibition between the 4th and 5th roots. These six roots are true seminal roots. Three additional roots, 7th, 8th, and 9th, can be formed in the sub-crown region. These roots are not formed from primordia of embryo (i.e., they are not seminal roots), and precautions have to be taken to avoid confusion with the seminal roots and true crown roots. [4]. A definition of “coleoptile nodal roots” has been suggested earlier for the designation of these roots [13]. True nodal roots emerge from closely spaced nodes starting from the first foliar node and form so-called “crown”, 5–10 roots per tiller and 10–25 roots per plant can be formed [14,15,16]. The appearance of true nodal roots starts with tillering within 3–4 weeks of seedling growth [17], but under optimal conditions, the initiation of nodal roots may begin as early as on day 11 [18].

Seminal and nodal roots differ in their anatomical structure; moreover, certain functional differences between them also exist. Seminal roots are the only roots responsible for the resource uptake in germinating seedlings, and therefore they are vitally important for seedling survival and early plant development [10]. Nodal roots usually appear soon after tillering, they provide plant anchorage and support nutrient and water uptake, especially during the reproductive stage of wheat growth [19]. Nodal roots usually lie within 40 cm of the soil surface, while seminal roots can grow deep down to 180 cm [20]. Seminal roots function throughout whole plant life extracting water and minerals from deeper soil levels [21,22], which is especially important under conditions of limited water availability. The larger number of seminal roots results in a larger root surface area, longer root system, and greater root biomass, as it is observed in domesticated wheat [2]. In addition to that, nodal roots lying in the topmost soil layer are more prone to damage under drought conditions, so a larger number of seminal roots may be associated with improved drought tolerance [23,24]. Indeed, a recent study examining the architecture of the root system in durum wheat demonstrates that genotypes with deep root systems have significantly increased grain yield and thousand kernel weight under water deficiency conditions in comparison to genotypes with shallow root system [25]. In another study, among different root traits of bread wheat seedlings tested in gel-chamber culture system, only the seminal root number trait correlated positively with grain yield of these cultivars measured in a field experiments [26]. Therefore, a large number of developed seminal roots is a potentially valuable agricultural trait, and the identification of genetic, physiological, and environmental factors regulating seminal roots development is an important task in applied plant biology. Even though many studies have been targeted toward the identification of the Quantitative Trait Loci (QTLs), determining the root architecture in wheat [7,8,10,26,27,28,29,30,31,32], the genetic factors determining the number of emerged seminal roots have not been identified so far. The number of developed seminal roots can vary significantly between individual plants of the same variety, and this parameter can be influenced by endogenous factors and environmental conditions. The data on physiological and environmental factors influencing the development of seminal roots are also quite limited. It has been demonstrated that the aging of seed may affect the ability to grow all seminal roots, while fresh seeds produce more seminal roots [4]. The evidence of a positive correlation between seed weight and root number has been presented [1]. It has also been demonstrated that the root number increases with the increase in embryo size [4,33,34] and is regulated by factors expressed in the embryo, not by endosperm [2]. Premature harvest, the concentration of sucrose (the major sugar transported to the embryo from endosperm during germination) in germination medium, and the temperature (only for several wheat varieties) have been shown to influence the appearance of seminal roots [4,35,36]. Growth of a sixth seminal root was shown to be initiated as a possible fallback under stress conditions after the death of previously formed seminal roots via yet not-known regulatory mechanisms [2,10].

Jasmonates are important signaling components coordinating plant responses under stress conditions [37]. Biosynthesis of jasmonates from α-linolenic acid is initiated in chloroplasts, where lipoxygenase-mediated oxidation leads to 13-HPOT (13-hydroperoxy-9,11,15-octadecatrienoic acid) formation [38]. 13-HPOT is converted to OPDA ((9*S*,13*S*)-12-oxo-phytodienoic acid) by the actions of enzymes allene oxide synthase and allene oxide cyclase [39]. OPDA is transported to peroxisomes, where OPDA reductase (OPR) reduces the double bond in the pentacyclic ring [40]. Then, as a result of three cycles of β-oxidation of the carboxylic acid side chain, jasmonic acid (JA) is formed [41]. The JA molecule can be further modified. The bioactive signaling ligand JA-isoleucine, regulating the majority of JA-dependent processes, is formed in cytoplasm. JA can be methylated to form volatile methyl jasmonate (MeJA) [42,43,44]. The jasmonates level in plant tissue is controlled by the sophisticated regulatory system including positive and negative feedback loops [45,46,47]. In monocots, especially in wheat, the jasmonate system remains poorly characterized [48]. In the present study, we demonstrate that jasmonates certainly influence the initiation and early growth of the sixth seminal root, and the effect strongly depends on concentration, duration of treatment, and unknown endogenous factors.

## 2. Results

### 2.1. Observations on the Early Development of the Root System

**Figure 2:**
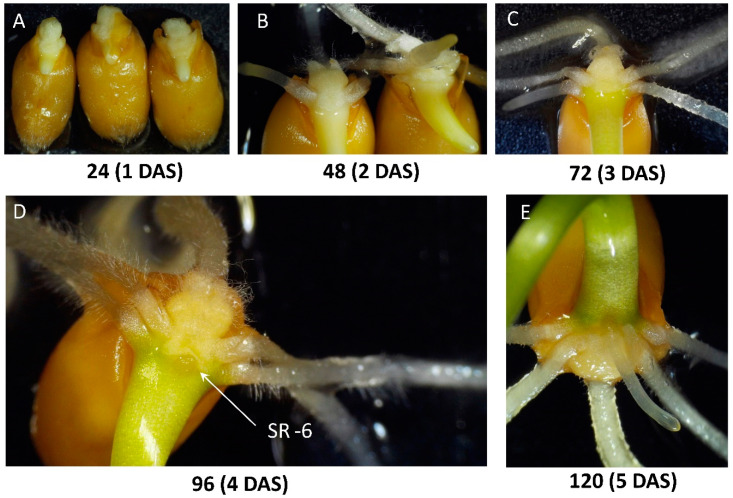
The growth of seminal roots within the first six days of seedling development. (**A**) Appearance of primary root; (**B**) seedling with three seminal roots; (**C**) seedling with five seminal roots; (**D**) appearance of SR-6; (**E**) seedling with six seminal roots. Numbers under the images indicate time in hours.

Initially, we performed microscopic observations of the early development of the root system in the studied plants (Figure 1 and Figure 2, Figures S1–S3). Figure 1A schematically illustrates the position of six seminal and three coleoptile nodal roots in the germinated seed. Seminal roots are represented by primary root (Root 1), first pair of seminal roots (Roots 2 and 3), second pair of seminal roots (Roots 4 and 5), and sixth seminal root (Root 6, SR-6) located above the primary root between Roots 4 and 5. Two coleoptile nodal roots (Roots 7 and 8) grow above the SR-6 slightly to the left and to the right of SR-6, and their appearance begins with the formation of small bulges at the base of the coleoptile (Figures S2–S4). Another coleoptile nodal root (Root 9) appears right above SR-6, breaking through the raptured surface layer of coleoptile tissue, and lying on a straight line with Root 1 and SR-6 (Figure S4E,F). A massive clump of coleorhiza cells surrounds the base of the primary root, and sometimes a layer of tissue forming cuffs around the base of four other seminal roots (Roots 2, 3, 4, and 5) can be observed (Figure 1B and Figure 2B–D, Figure S1A). It looks like SR-6 starts growing under the coleorhiza tissue, and when it emerges, it pushes and bends back the layer of coleorhiza cells, at least in studied wheat varieties (Figure 1C,D, Figure S2). SR-6 usually appears on day five and can be identified based on its position above the primary root between Root 4 and Root 5, contacting or pushing coleorhiza (Figure 1D and Figure 2D, Figure S2A,B). As seen in Figure 2, showing the first five days of seeds germination, the formation of the SR-6 is obvious on the fifth day after sowing, but the employment of a digital dissecting microscope allows us to distinguish this root already on day four. Coleoptile nodal Roots 7 and 8 can grow regardless of the presence or absence of SR-6 (Figure S3A–C), and sometimes SR-6 appears when Roots 4 and 5 are absent (Figure S3D). Interestingly, Root 9 was observed only when the 6th root was present. While SR-6 can be reliably identified at the very early growth stages, later (in 7–10 days) such identification becomes difficult due to the appearance of coleoptile nodal roots, bending of grown roots, and formation of bundles (Figures S3B and S4F). Thus, the necessary information has been obtained to ensure the accuracy of SR-6 identification during the early stages of root system development.

### 2.2. Exogenous Treatment of Non-Transgenic Seeds with MeJA

**Figure 3:**
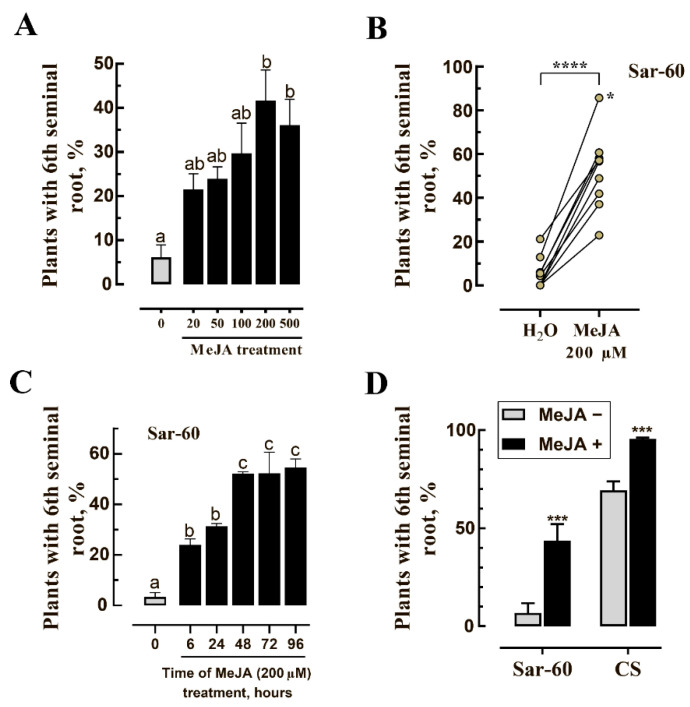
Jasmonate treatment stimulates the development of SR-6. (**A**) The percentage of Sar-60 seedlings with developed SR-6 after 96-h treatment with different concentrations of MeJA. The number of plants with SR-6 were scored on the fifth DAS when plants were treated with 0–100 μM concentration of MeJA, and on the sixth DAS when plants were treated with 200–500 μM MeJA; (**B**) pairwise comparison of the plants treated for 96 h with 200 μm MeJA with the corresponding control (not treated with MeJA), the star indicates the experiment with freshly collected seeds; (**C**) the dependence of the frequency of SR-6 appearance on the time of treatment with MeJA; (**D**) the effect of MeJA treatment on the appearance of SR-6 in Sar-60 and ‘Chinese Spring’ (CS) plants. Different letters above the bar graphs in (**A**) and (**C**) indicate statistical difference between treatments determined in one-way analysis of variance (ANOVA) followed by Tukey’s post hoc test (*p* ≤ 0.05); “*” in (**B**) indicates experiment with freshly harvested seeds; “****” indicates statistical difference between treatments determined by Student’s t-test (*p* ≤ 0.0001); in (**D**) “***” indicates statistical difference between treatments determined by Student’s t-test (*p* ≤ 0.001).

Different molecular mechanisms could be responsible for the initiation of root growth and the regulation of root growth as such. In the present work, we studied the effect of jasmonates on the sixth seminal root appearance without considering the rate of SR-6 growth and even took into account the roots that appeared as small bulges similar to shown in (Figure S1B). Several concentrations of MeJA were applied to the Petri dishes with germinating seeds, and plants with emerged SR-6 were scored. It is known that when applied exogenously, MeJA easily crosses the cell membrane and gets de-methylated in tissue to produce free jasmonic acid [49]. In agreement with previously reported data [50,51,52], MeJA significantly suppresses root growth (Figure S5). Due to this fact, the presence or absence of SR-6 on MeJA-treated plants was confirmed on the sixth day after sowing. As well as the obvious suppression of seedling growth, the characteristic effect of jasmonate such as bending of coleoptiles and roots [53] was observed (Figure S6). The trend of increase in the number of plants with SR-6 was observed already after treatment of seeds with 20 μM MeJA, however, the significant increase in the number of seedlings with six seminal roots was achieved only after the treatment of seeds with 200 μM MeJA, when the rate of SR-6 appearance was 41.7% (Figure 3A). Further increase in MeJA concentration does not increase the stimulating effect of treatment on SR-6 appearance. The results of multiple experiments on seed treatment with 200 μM MeJA (Figure 3B) demonstrate that the frequency of the sixth root emergence may vary noticeably among different seed batches, probably due to the difference in the seeds age and their ontological history, but the stimulating effect of MeJA on the SR-6 appearance is always obvious. An especially strong effect of MeJA was observed in experiments with freshly collected seeds (three days after harvesting) when the SR-6 appearance rate in MeJA-treated seeds was higher than 80% (corresponds to the highest point among dots representing MeJA-treated samples in Figure 3B, marked with a star). It should be noted that the germination rate of freshly collected seeds not treated with MeJA was low (about 36%), and interestingly enough, in addition to the stimulating effect on SR-6 appearance, MeJA treatment led to an increase in the percentage of germinated seeds (Figure S7).

The duration of seeds’ exposure to MeJA also influences the rate of SR-6 appearance. Six hours of exposure to the 200 μM MeJA led to a significant increase in the percentage of plants with SR-6 (above 20%) (Figure 3C). However, more than half of the plants possessed SR-6 if the incubation time was increased up to 48 h. Further increase in the incubation time did not enhance the stimulating effect, and about 50% of germinated plants possessed SR-6 after exposure of seeds to exogenous MeJA for 72 and 96 h.

Based on the information available in the literature, plants of the ‘Chinese Spring’ (CS) variety are characterized by the ability to produce a higher number of seminal roots compared to other studied varieties [4,6,7,54,55]. CS seedlings were shown to have on average 5.8 seminal roots [4]. This fact prompted us to look at the formation of SR-6 in CS plants and to study the effect of MeJA on this process. Even when not treated with MeJA, more than 70% of CS seeds produce six seminal roots, and MeJA treatment increases the number of plants with SR-6 up to 100% (Figure 3D), demonstrating that the observed stimulating effect of MeJA on SR-6 formation is not limited to ‘Saratovskaya-60’ (Sar-60), but is also manifested in other hexaploid varieties.

### 2.3. Analysis of Transgenic Wheat Plants Overexpressing AtOPR3

To study the effect of endogenously produced jasmonates on the formation of SR-6, we analyzed transgenic wheat plants of Sar-60 variety overexpressing the gene coding for the peroxisome-localized enzyme of jasmonate biosynthesis pathway, 12-oxo-phytodienoate reductase (AtOPR3) from *Arabidopsis thaliana* [56]. AtOPR3 gene codes for the peroxisome-localized enzyme controlling the levels of major metabolites of the jasmonate pathway, the upstream substrate OPDA, and the downstream products of the pathway, including JA and its derivatives [40,57,58,59]. Transgenic lines Tr-3 and Tr-18 increased basal levels of JA determined in the second leaves of transgenic plants at the four-leaf stage. These plants are characterized by delayed germination, they display slower growth, later flowering, and delayed senescence [56]. In other independent transgenic lines, on the contrary, basal JA level is significantly decreased in comparison with non-transgenic control plants, the plants germinate earlier, grow faster, display earlier flowering, and senescence (brightly manifested in Tr-20 line).

**Figure 4:**
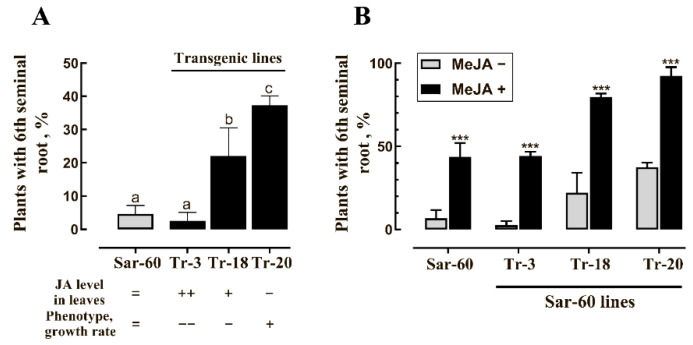
AtOPR3 (12-oxo-phytodienoate reductase) overexpression affects the frequency of SR-6 appearance. (**A**) Percentage of plants with the developed SR-6 in non-transgenic Sar-60 and transgenic lines Tr-3, Tr-18, and Tr-20 differing in endogenous jasmonates levels (“++”, increased; “+”, slightly increased, “−“, decreased in comparison to Sar-60) and growth phenotype (“−−”, delayed growth; “−”, slightly delayed growth, “+“, growing faster in comparison to Sar-60), as indicated below bar graphs); (**B**) response of transgenic plants with altered jasmonate levels to exogenous MeJA treatment. Different letters above bar graphs in (**A**) indicate statistical difference between lines determined in one-way analysis of variance (ANOVA) followed by Tukey’s post hoc test (*p* ≤ 0.05); “***” in (**B**) indicates statistical difference between treatments determined by Student’s t-test (*p* ≤ 0.001).

The decrease in endogenous jasmonate level resulted from the overexpression of jasmonate biosynthesis pathway genes has been observed in several studies [58,60,61,62], and the possible mechanisms associated with the negative feedback loops, regulating jasmonate levels, or the inhibition of enzymatic activity of OPR due to the dimerization of proteins have been previously discussed [45,46,47,56,63]. Unexpectedly, the rate of SR-6 formation did not correlate with the level of endogenous jasmonic acid levels (Figure 4). The seeds of Tr-3 plants with the highest level of JA in leaves produced the lowest number of SR-6, while the rate of SR-6 formation was highest among the seeds of Tr-20 plants with the lowest JA level in leaves. Exogenous MeJA treatment stimulates the formation of SR-6 in all transgenic plants, but in the case of Tr-20, almost 100% of seedlings formed SR-6 after the treatment with MeJA. Importantly, lower concentrations of MeJA were needed to induce the stimulative effect on SR-6 induction in Tr-20; 95% of Tr-20 seeds treated with 20 μM MeJA produce SR-6 (Figure S8).

## 3. Discussion

There remain significant gaps in our knowledge about the early development of monocotyledonous seedlings, some basic aspects require further clarifications and accurate description [64], even when it comes to such an important crop as wheat. In this work, we closely observed the early development of the root system in bread wheat and discovered the effect of the stress hormone jasmonate on the formation of the sixth seminal root. Seminal roots appear from the very beginning of plant development, actively function throughout the whole plant life, and under certain conditions, such as drought or death of other roots, represent the only roots plant rely on for water and mineral uptake [21,22]. The mechanisms regulating the development of the sixth seminal root were unknown. Available evidence suggests that genetic and environmental factors influence the formation of this root [2]. The physiological plasticity in young seedlings, including the ability to maintain a root in dormancy or initiate its growth when needed, is an important factor determining the ability to adapt to changing environmental conditions. Bread wheat cultivars of Saratov selection (steppe ecotype) display a relatively high rate of sixth root formation. Possibly, this characteristic is predetermined by the adaptation to the necessity to grow fast at early developmental stages to overcome the effect of early spring drought often occurring in the Russian steppe zone [65].

The involvement of jasmonates in the regulation of root growth was previously demonstrated in multiple studies, and JAs were suggested to be a potential signaling hormone in shaping root architecture (reviewed in [66]). In most cases, the regulatory role of JAs was manifested as suppression of root growth. Inhibition of primary root growth by jasmonates was shown in *Arabidopsis* [44,66,67,68,69,70,71,72,73], rice [74], tomato [52], sunflower [75], and it was demonstrated that this function is implicated through the inhibition of cell division and elongation [50,51,52]. While the jasmonate effect on the primary root is quite unambiguous, the effect of this hormone on other types of roots is less understood. JA displayed an opposite effect on the elongation of lateral roots in *Arabidopsis* and sunflower: promoted in *Arabidopsis* and suppressed in sunflower [50,75]. The available data on the effects of JA on adventitious roots formation are also contradictory. In petunia cuttings, the decrease in the levels of JA and its bioactive conjugate JA-isoleucine resulted in the reduced formation of adventitious roots [76]. JAs have a negative effect on the formation of the adventitious roots in de-etiolated *Arabidopsis* seedlings [77], but stimulate this process in tobacco dark-grown thin cell layers in the presence of indole-3-butyric acid and kinetin, with an increase in endogenous JA levels preceding the formation of adventitious roots [49,78]. Continuous treatment with a low concentration of OPDA, JA, or MeJA inhibited the adventitious roots formation in several plants, including petunia leaf cuttings, *Arabidopsis*, and *Bupleurum kaoi* [76,77,79]. While long-term treatment of plants with JA inhibited the formation of adventitious roots, under certain conditions, JA together with ethylene had an opposite effect through the triggering the expression of genes involved in the biosynthesis of indole-3-acetic acid (IAA), major auxin responsible for the regulation of root growth [80,81,82]. This may suggest that a certain threshold of JAs is required for adventitious roots initiation, but when present continuously, JAs inhibit this process.

Auxin is a major hormone regulating adventitious and lateral root growth [80,83]. The studies performed on mutants defective in auxin biosynthesis, transport, or signaling demonstrate that this hormone inhibits root elongation and promotes lateral and crown root initiation in monocots [84,85]. JA-auxin crosstalk could be a possible mechanism of JA involvement in the regulation of SR-6 formation. The multiple pieces of evidence demonstrate the interaction of JA with auxin at different levels, including the JA involvement in the modulation of auxin biosynthesis, transport, and signaling pathways [66,77,86,87,88]. In previous studies, it has been suggested that in monocotyledons the jasmonates inhibit the cell wall polysaccharide synthesis and regulate intracellular pH [89,90,91], wherein JA and IAA have been shown to act antagonistically [92,93]. Furthermore, jasmonates can modulate the activity of potassium channels [94] that are involved in auxin-dependent growth and gravitropic responses [95]. In addition, jasmonate might reduce auxin responsiveness through the recruitment of signaling factors such as auxin resistant 1 (AXR1) and, through this, interfering with the auxin signaling [96]. Other possible mechanisms underlying jasmonate-auxin crosstalk in the regulation of root system development have been previously discussed [86]. The role of jasmonate as an antagonist of auxin in gravitropic responses in rice was also demonstrated [53]. Interestingly, the formation of the JA gradient within tissues was accompanied by a gradient of the OPDA, the substrate of JA biosynthesis, in the opposite direction, suggesting a gradient in OPR enzyme activity.

The generated data demonstrate that jasmonates, endogenously produced or exogenously applied, influence the formation of the sixth seminal root, but this effect manifestation strongly depends on the additional factors. The possible decrease in endogenous jasmonate levels responsible for the observed effect should not be ruled out. It has been previously convincingly demonstrated on citrus plants that exogenously applied MeJA suppresses the biosynthesis of endogenous jasmonates through the inhibition of the activities of key enzymes of the biosynthetic pathway, especially 12-oxo-phytodienoic acid reductase (OPR) [97].

The present study describing the effect of jasmonates on the sixth seminal root formation in wheat provides a valuable instrument for the further understanding of the jasmonates’ role in root system formation and hormonal crosstalk responsible for this regulation.

## 4. Materials and Methods

### 4.1. Plant Material and Growth Conditions

The non-transgenic seeds of spring bread wheat (*Triticum aestivum* L., 2n = 6x = 42) varieties ‘Saratovskaya-60’ (noted as Sar-60 thereafter) and ‘Chinese Spring’ (noted CS thereafter) were used in the study. Additionally, the transgenic seeds of transgenic lines of Sar-60 constitutively expressing the *Arabidopsis 12-oxophytodienoate reductase 3* gene (*AtOPR3*) under the control of the maize *ubiquitin* (*Ubi1*) promoter [56] were used. Stock plants were grown in pots and maintained in a greenhouse with an environmental growth regime of 25 ± 2 °C, light, 16 h, or 20 ± 2 °C, dark, 8 h with additional lighting when needed to supply the light intensity of 150 photons µmol m^−2^·s^−1^. To study the development of seminal roots, mature seeds were placed on two layers of wet filter paper in square Petri dishes (10 by 10 cm, Sarstedt, Nümbrecht, Germany), 30–40 seeds per plate. Before planting, seeds were surface sterilized with 2% sodium hypochlorite solution for 20 min and washed with sterile distilled water for four 5-min periods. Seeds were grown under dim light (about 5 μmol photons m^−2^·s^−1^) emitted with 10 Wt lamp (LED, Uniel, Chernogolovka, Russia), under 12 h:12 h light:dark cycles.

To evaluate the effect of exogenous methyl jasmonate treatment, the seeds germinating on filter paper in Petri dishes were wetted by 15–17 mL of the aqueous solution of methyl jasmonate (Sigma-Aldrich, St. Louis, MO, USA) of given concentration or sterile water (for control) on the first day of germination. The number of roots was counted five days after sowing, except for experiments with 100–500 μM MeJA, when the number of roots was counted on the sixth day after sowing. For long-term experiments (up to 14 days), seeds were transferred to deeper plastic containers with water in the bottom to keep roots covered with water.

For observation and imaging of germinating seeds, digital dissecting microscope Andonstar ADSM301 (Andonstar, Shenzhen, China) was used.

### 4.2. Data Analysis

The experiments were carried out at least three times. To determine significant differences between samples and treatments, one-way analysis of variance (ANOVA) have been performed. This was followed by Student’s *t*-test (for pairwise comparison) or Tukey’s post hoc test (for multiple comparisons) when significant differences (*p* ≤ 0.05) were found. Asterisks and letters were used in graphs to indicate statistically significant differences.

## Data Availability

Data is contained within the present article and supplementary material.

## Supplementary Materials

The following are available online at https://www.mdpi.com/2223-7747/10/2/219/s1. Figure S1. Seedlings without (A) and with (B) SR-6. SR-6 at this stage is visible as a small bulge. Figure S2. Characteristics of the SR-6. (A,B) 10-day-old wheat seedlings with 6 seminal and two coleoptile roots. SR-6 is positioned on the same horizontal line with 4th and 5th seminal roots, and the characteristic displacement of the coleorhiza is visible. Figure S3. Variations in the root system. (A) A wheat seedling at 11 DAS with 7 roots, but lacking the SR-6, representing a situation where coleoptile nodal root can be mistakenly taken as the SR-6; (B) Formation of a tangled bundle of roots that makes it difficult to identify the individual roots. (C) Wheat seedling at 11 DAS with 8 roots, including the SR-6; (D) A rare case when in a 6-day-old Sar-60 seedling, the development of the SR-6 began earlier than the 2nd pair of seminal roots (Roots 4 and 5). Figure S4. The sites and the order of the coleoptile nodal roots appearance. (A) 8-day-old wheat seedling with characteristic swellings on the sites where 7th and 8th coleoptile nodal roots later will appear; (B) 7th and 8th roots germination; (C) An example when one coleoptile nodal root developed faster than others; (D–F) Development of the 9th coleoptile nodal root at the 11th–14th DAS in seedling with developed (D,F) and undeveloped (E) 7th and 8th nodal roots. In all cases, the position of the 9th nodal root was above the SR-6 on the same line with the primary root and SR-6, and the characteristic tear on the surface of the stem at the site of 9th root emergence was observed. Figure S5. Effect of the duration of treatment with 200 μM MeJA on the growth of wheat seedlings. Plants of Sar-60 were treated with 200 μM MeJA for the indicated period, pictures were taken at 5th DAS. Figure S6. Typical view of wheat seedlings treated with 200 μM MeJA during 96 h on the 6th DAS. This time point is good for the scoring of plants with SR-6 after the treatment with a high concentration of MeJA. Characteristic bending of roots and coleoptile and root thickening were observed. Figure S7. Effect of MeJA on the germination of freshly harvested seeds (within a week after harvest). The light gray-colored bar represents germinated by 5th DAS seeds not treated with MeJA. 20 seeds (out of 31 untreated) that had not germinated by 5th DAS were then treated with 200 μM MeJA, and the sum of seeds germinated before and after treatment is represented with the dark gray bar. The germination rates for seeds treated with different concentrations of MeJA for 96 h from the very beginning of the experiment are shown in black. Figure S8. The higher sensitivity of Tr-20 to MeJA. For each MeJA concentration, 35–40 seeds were used. Plants were scored on 5th DAS for control and 20–100 μM MeJA-treated seeds and on 6th DAS for seeds treated with higher MeJA concentrations.
