# Supplementary material for: Regulation of Sixth Seminal Root Formation by Jasmonate in Triticum aestivum L
Source: Plants (Basel). 2021 Jan 23;10(2):219. doi: 10.3390/plants10020219 (PMC7911905; doi:10.3390/plants10020219)
Supplement: Supplementary file 1 [file plants-10-00219-s001.pdf]

plants

MDPI

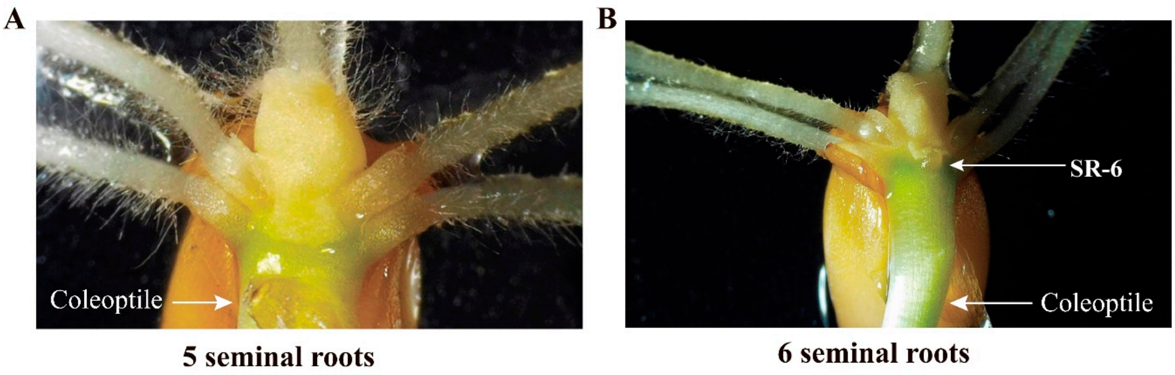

Figure S1. Seedlings without (**A**) and with (**B**) SR-6. SR-6 at this stage is visible as a small bulge.

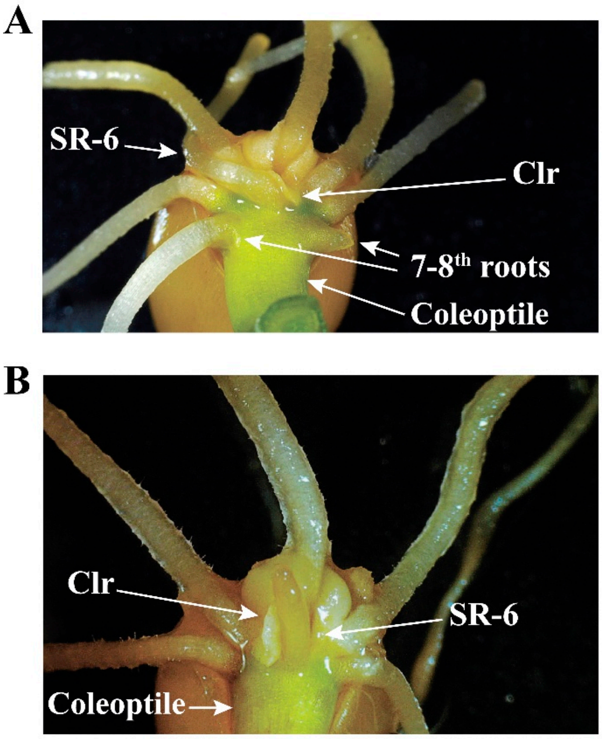

Figure S2. Characteristics of the SR-6. (**A**,**B**) 10-day-old wheat seedlings with 6 seminal and two coleoptile roots. SR-6 is positioned on the same horizontal line with 4th and 5th seminal roots, and the characteristic displacement of the coleorhiza is visible.

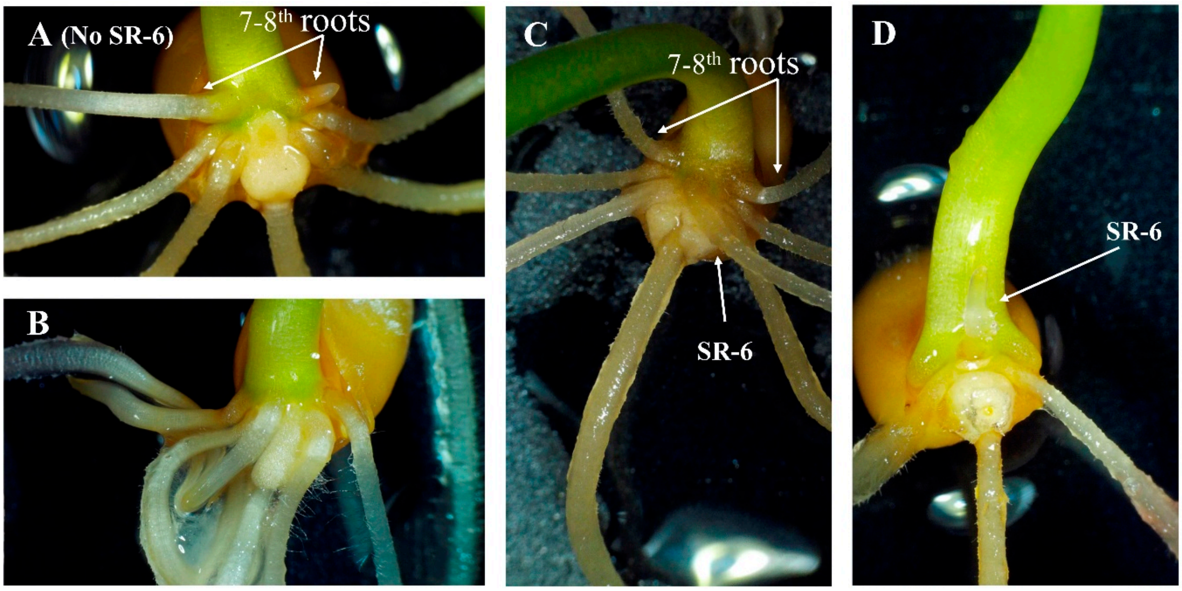

Figure S3. Variations in the root system. (**A**) A wheat seedling at 11 DAS with 7 roots, but lacking the SR-6, representing a situation where coleoptile nodal root can be mistakenly taken as the SR-6; (**B**) Formation of a tangled bundle of roots that makes it difficult to identify the individual roots. (**C**) Wheat seedling at 11 DAS with 8 roots, including the SR-6; (**D**) A rare case when in a 6-day-old Sar-60 seedling, the development of the SR-6 began earlier than the 2nd pair of seminal roots (Roots 4 and 5).

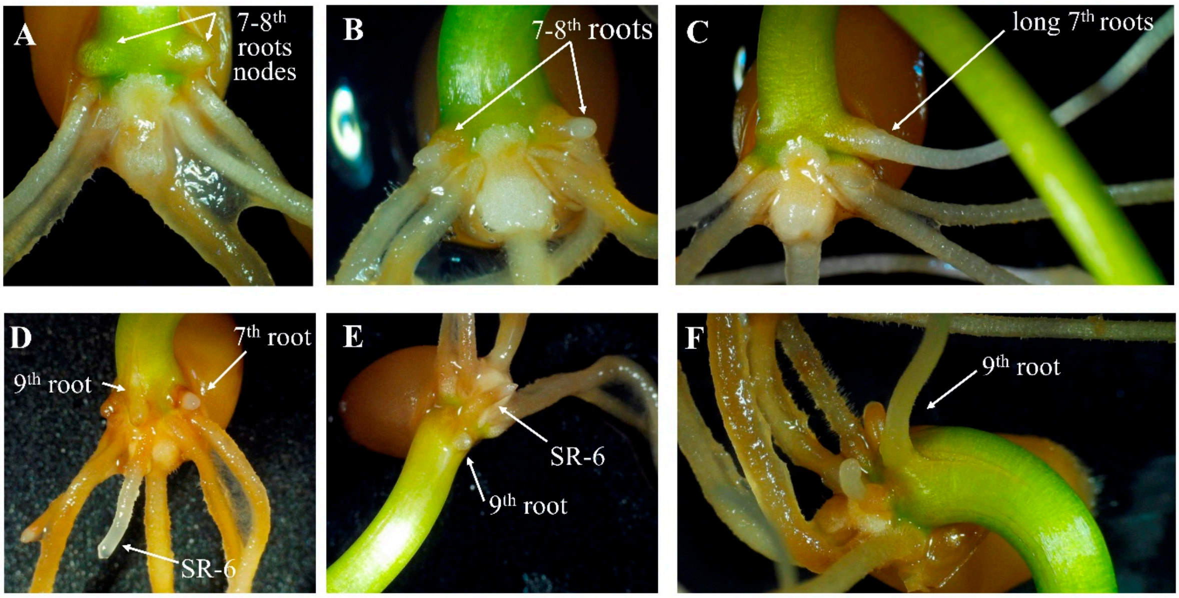

Figure S4. The sites and the order of the coleoptile nodal roots appearance. (**A**) 8-day-old wheat seedling with characteristic swellings on the sites where 7th and 8th coleoptile nodal roots later will appear; (**B**) 7th and 8th roots germination; (**C**) An example when one coleoptile nodal root developed faster than others; (**D–F**) Development of the 9th coleoptile nodal root at the 11th–14th DAS in seedling with developed (**D**,**F**) and undeveloped (**E**) 7th and 8th nodal roots. In all cases, the position of the 9th nodal root was above the SR-6 on the same line with the primary root and SR-6, and the characteristic tear on the surface of the stem at the site of 9th root emergence was observed.

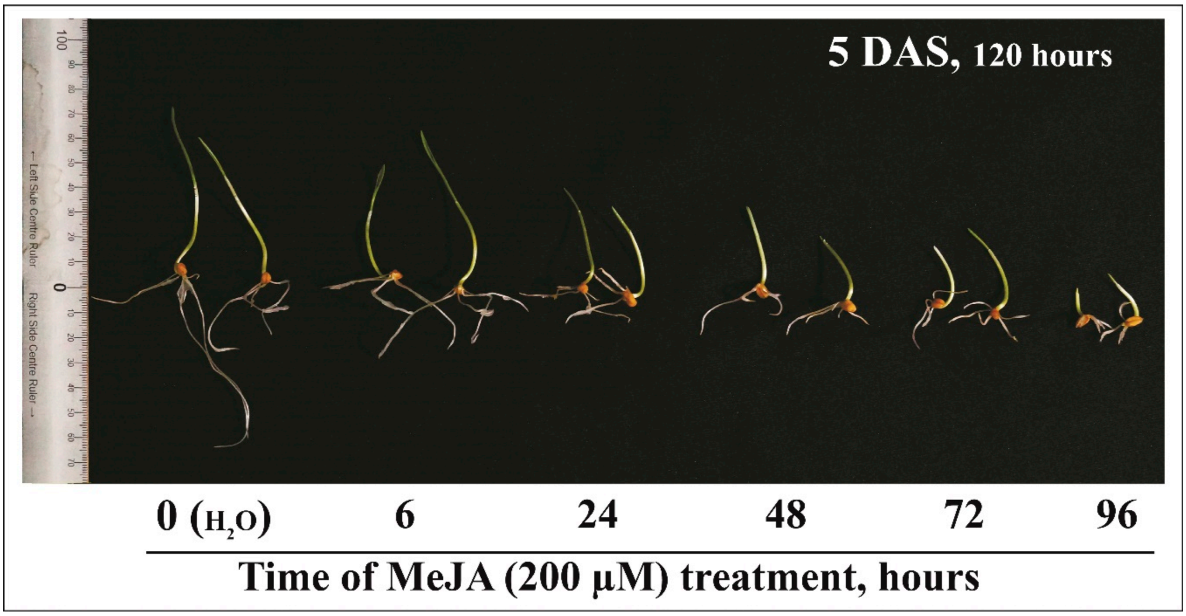

Figure S5. Effect of the duration of treatment with 200 µM MeJA on the growth of wheat seedlings. Plants of Sar-60 were treated with 200 µM MeJA for the indicated period, pictures were taken at 5th DAS.

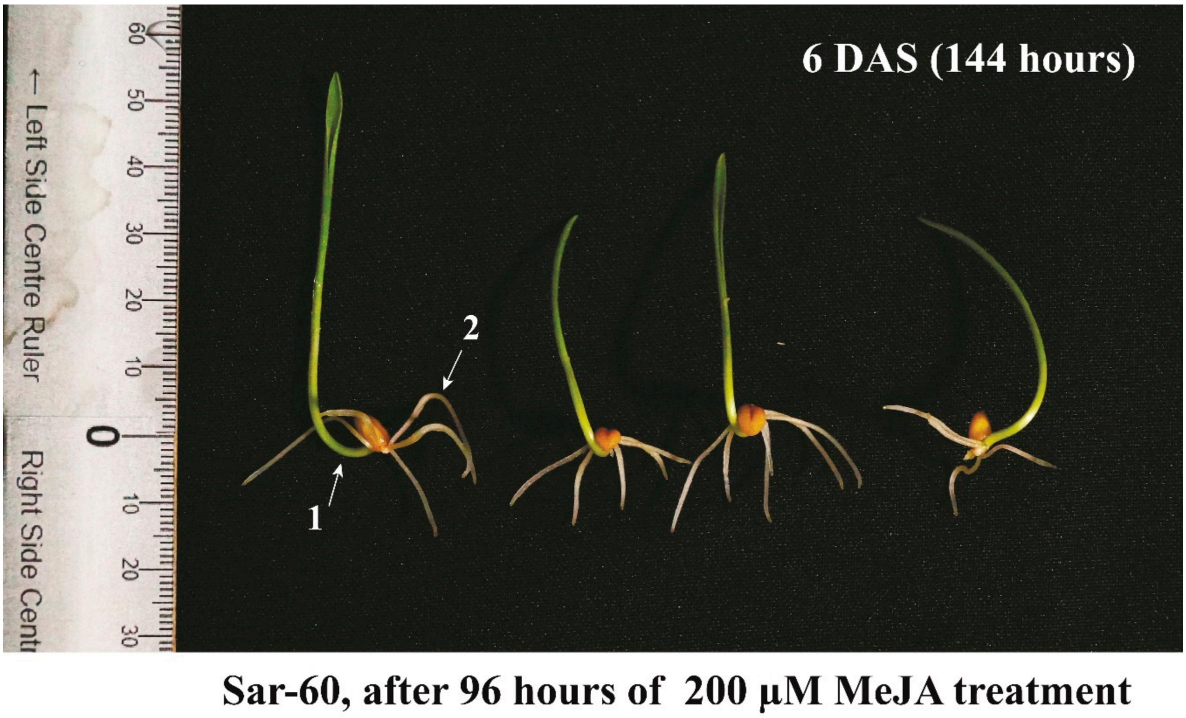

Figure S6. Typical view of wheat seedlings treated with 200 µM MeJA during 96 h on the 6th DAS. This time point is good for the scoring of plants with SR-6 after the treatment with a high concentration of MeJA. Characteristic bending of roots and coleoptile and root thickening were observed.

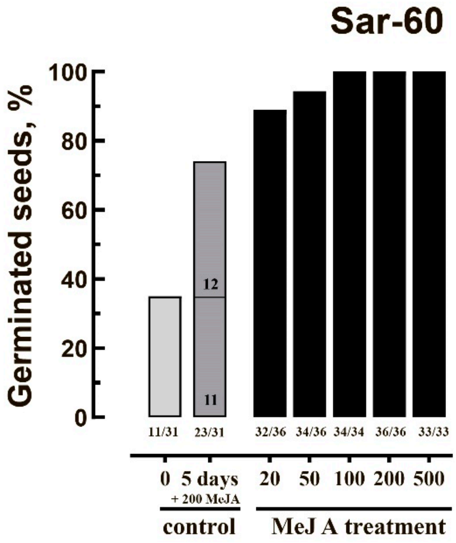

Figure S7. Effect of MeJA on the germination of freshly harvested seeds (within a week after harvest). The light gray-colored bar represents germinated by 5th DAS seeds not treated with MeJA. 20 seeds (out of 31 untreated) that had not germinated by 5th DAS were then treated with 200 µM MeJA, and the sum of seeds germinated before and after treatment is represented with the dark gray bar. The germination rates for seeds treated with different concentrations of MeJA for 96 h from the very beginning of the experiment are shown in black.

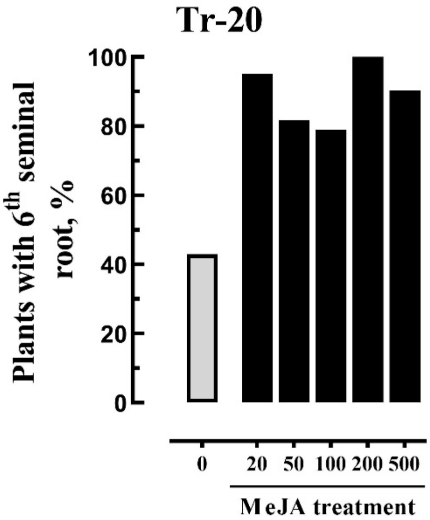

Figure S8. The higher sensitivity of Tr-20 to MeJA. For each MeJA concentration, 35–40 seeds were used. Plants were scored on 5th DAS for control and 20–100 µM MeJA-treated seeds and on 6th DAS for seeds treated with higher MeJA concentrations.
